# The Impact of Body Mass Index and Nutritional Status on Cardiac Electrophysiological Balance Using ICEB and ICEBc: A Cross-Sectional Approach

**DOI:** 10.3390/jcdd13030109

**Published:** 2026-02-26

**Authors:** Fethullah Kayan, Ömer Faruk Alakuş, Mihriban Elçiçek, Serdar Soner, Cansu Öztürk, Geylani Güleken, Ihsan Solmaz

**Affiliations:** 1 Department of Cardiology, Saglik Bilimleri University Diyarbakir Gazi Yasargil Training and Research Hospital, 21070 Diyarbakir, Turkey; drserdar_89@hotmail.com (S.S.); dr.cansu.ozturk@gmail.com (C.Ö.); gguleken217@gmail.com (G.G.); 2 Department of Internal Medicine, Saglik Bilimleri University Diyarbakir Gazi Yasargil Training and Research Hospital, 21070 Diyarbakir, Turkey; omerfaruk01@gmail.com (Ö.F.A.); ihsan2157@gmail.com (I.S.); 3 Department of Nutrition and Dietetics, Tekirdağ Namık Kemal University, 59020 Tekirdağ, Turkey

**Keywords:** cardiac electrophysiological balance, ICEB, ICEBc, obesity, body mass index, nutritional status, GNRI, HALP score, ventricular repolarization, electrocardiography

## Abstract

Background: The Index of Cardiac Electrophysiological Balance (ICEB) has emerged as a electrocardiographic marker reflecting the equilibrium between ventricular depolarization and repolarization. Although obesity is known to alter cardiac electrophysiology, the combined influence of body mass index (BMI) and objective nutritional status on ICEB and its heart rate-corrected form (ICEBc) remains insufficiently defined. This study aimed to investigate the associations between BMI categories, nutritional status, and cardiac electrophysiological balance. Methods: This cross-sectional study included 591 adult patients classified as normal-weight, overweight, or obese according to BMI. Electrophysiological assessment of ICEB (QT/QRS) and ICEBc (QTc/QRS) values was calculated from standard 12-lead electrocardiogram recordings. Participants’ nutritional status was analyzed using validated clinical indices such as the Prognostic Nutritional Index (PNI), Controlling Nutritional Status (CONUT), Geriatric Nutritional Risk Index (GNRI) and Hemoglobin–Albumin–Lymphocyte–Platelet (HALP) score. Results: According to the results, both ICEB and ICEBc showed significant differences among BMI categories (*p* < 0.001). ICEB/ICEBc exhibited a non-linear distribution. The ICEB/ICEBc values were found to be minimum in the normal weight group at 4.22 ± 0.54/4.87 ± 0.66 and maximum in the obese group at 4.27 ± 0.51/4.99 ± 0.59. The ICEB/ICEBc value closest to the optimal physiological limits was found in the overweight group at 4.04 ± 0.53/4.59 ± 0.58. Higher ICEBc quartiles were accompanied by increased GNRI (120.9 ± 13.7, 129 ± 15.1, 130.5 ± 16.3, 131.8 ± 17.6, *p* < 0.001)and decreased HALP scores (59.7 ± 24.4, 56.1 ± 25.3, 55.2 ± 25.9, 51.1 ± 19.4, *p*: 0.025). Conclusion: The association between BMI and cardiac electrophysiological balance is non-linear and appears to be modulated by nutritional and inflammatory status. ICEBc may represent a more sensitive marker than ICEB for detecting subtle electrophysiological alterations related to obesity.

## 1. Introduction

Cardiovascular diseases(CVDs), despite medical and technological advances in prevention and treatment methods, are still the leading cause of morbidity and mortality worldwide [1]. The recent literature increasingly emphasizes that maintenance of myocardial electrophysiological stability is a critical determinant in the pathogenesis of ventricular arrhythmias, alongside traditional risk markers [2]. In particular, abnormal changes in ventricular depolarization and repolarization processes are thought to cause malignant arrhythmias, which can be fatal.

In this context, the Cardiac Electrophysiological Balance Index (ICEB), calculated as the ratio of the QRS duration, symbolizing ventricular depolarization, and the QT interval, reflecting repolarization, is a non-invasive electrocardiographic parameter [3]. Conceptually, ICEB is a mathematical combination of conduction velocity and refractory period and reflects the myocardial excitation wavelength (λ).

It is thought that bidirectional deviations (increase or decrease) in this balance may increase the sensitivity to ventricular arrhythmogenesis through different pathophysiological pathways [4]. Consequently, ICEB possesses the potential to reflect the cardiac electrophysiological state more comprehensively than the evaluation of QT or QRS duration alone [5].

Obesity, whose prevalence is increasing on a global scale, is an important risk factor for the occurrence of cardiovascular diseases, which progresses with systemic and chronic subclinical inflammation and affects cardiac electrophysiology [6]. The interaction between Body Mass Index (BMI) and ventricular depolarization–repolarization abnormalities has been the subject of scientific research for a long time. However, according to some results, it has been observed that overweight individuals with a BMI of 25 and under 30 may paradoxically be associated with more positive outcomes in some cardiovascular conditions. This phenomenon, termed the obesity paradox, has recently garnered significant attention among clinicians [7]. However, the projections of this paradoxical relationship on cardiac electrophysiological stability and the modulatory role of nutritional status in this interaction have not yet been clarified in the literature.

Nutritional status is one of the main determinants in maintaining cardiovascular health and determining clinical prognosis [8]. It is well-established that individual biochemical parameters, such as serum albumin or hemoglobin, may be insufficient to comprehensively reflect an individual’s metabolic reserve, inflammatory burden, and immunological status [9]. Therefore, multidimensional nutritional indices, such as the Geriatric Nutritional Risk Index (GNRI), the Hemoglobin–Albumin–Lymphocyte–Platelet (HALP) score, the Controlling Nutritional Status (CONUT) score, and the Prognostic Nutritional Index (PNI), are increasingly utilized in clinical research to assess nutritional and inflammatory status in an integrated manner [10]. Previous studies have demonstrated that, in particular, GNRI and HALP scores can predict mortality and morbidity in both chronic cardiovascular diseases and acute cardiac events [11,12]. Nutritional and inflammatory disorders can affect cardiac electrophysiology through a multitude of interconnected mechanisms. Malnutrition and systemic inflammation can affect ventricular repolarization dynamics and QT duration, particularly by disrupting potassium, magnesium, and calcium balance [13]. Chronic inflammatory activation promotes myocardial fibrosis and structural remodeling, which can directly affect QRS duration by altering conduction velocity and refractoriness. Furthermore, inflammatory cytokines such as interleukin-6 and tumor necrosis factor-α have been shown to contribute to repolarization abnormalities and susceptibility to arrhythmias by modulating ion channel function and autonomic tone [14]. These processes can disrupt the depolarization–repolarization balance reflected by ICEB and ICEBc.

Despite the potential of ICEB in assessing ventricular arrhythmia risk, studies examining the combined impact of different BMI categories and objective nutritional indices on ICEB—and its heart rate-corrected form, ICEBc—are quite limited [15]. The modulating role of nutritional status in the interaction between adiposity and cardiac electrophysiological stability has not been the subject of sufficient systematic reviews. The combined analysis of electrophysiological data and nutritional dynamics may be of critical importance for the proactive management of ventricular arrhythmias in overweight and obese individuals. It is thought that this synergy will provide a beneficial clinical vision that enables patient-based preventive approaches.

The purpose of this study is to examine the relationship between BMI and ICEB/ICEBc in groups of normal weight, overweight, and obese individuals in different BMI categories, to reveal the relationships between ICEB/ICEBc and various nutritional risk indices.

## 2. Materials and Methods

### 2.1. Study Design and Population

**Figure 1:**
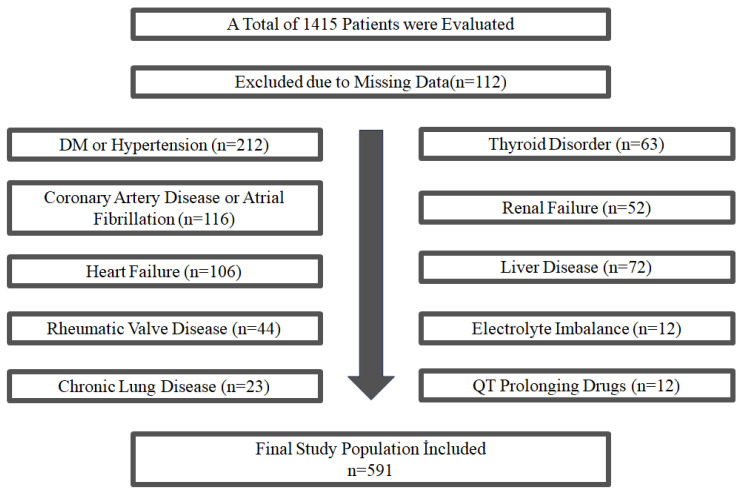
Flowchart of patient selection and exclusion criteria.

Our study included adult patients who were consulted with non-specific symptoms at the cardiology, obesity, and internal medicine outpatient clinics of Gazi Yaşargil Training and Research Hospital, Health Sciences University, between January 2023 and June 2023. Initially, 1415 patients were screened; after applying the exclusion criteria (as shown in Figure 1), a total of 591 patients were included in the final analysis. Patients were divided into three groups according to their BMI values: Normal weight (18.5–24.9 kg/m^2^, *n* = 91), Overweight (25.0–29.9 kg/m^2^, *n* = 122), and Obese (≥30.0 kg/m^2^, *n* = 378). To examine potential curvilinear relationships, BMI was analyzed as a continuous variable using second-order regression models including BMI^2^. Additionally, four-node constrained cubic spline modeling was performed to evaluate potential nonlinear relationships between BMI and ICEB/ICEBc. Adjusted spline models were constructed, including age, heart rate, GNRI, albumin, and hemoglobin. The study protocol (409-28 March 2025) was approved by the Diyarbakır Gazi Yaşargil Training and Research Hospital Ethics Committee and conformed to the standards set in the Declaration of Helsinki.

### 2.2. Electrocardiography and Laboratory

Standard 12-lead ECGs were recorded using a Schiller Cardiovit At-102 G2 (25 mm/s, 10 mm/mV) device (Schiller, Baar, Switzerland). The digital analysis of the ECG recordings was performed independently by two cardiologists using Adobe Photoshop (Adobe Inc., San Jose, CA, USA), version 12.0]. To minimize measurement errors, three separate measurements were taken for each lead, and their arithmetic mean was recorded. ECG measurements were performed manually by two independent cardiologists who were blinded to clinical data. QT and QRS intervals were measured from three consecutive beats in lead II and, when necessary, confirmed in lead V5. The average value was recorded for analysis. In cases of discrepancy, measurements were reviewed jointly, and consensus was achieved. QTc was initially calculated using the Bazett formula. As a sensitivity analysis, QTc was also recalculated using the Fridericia correction, yielding ICEBcF (QTcF/QRS) [16]. All individuals included in the study were in sinus rhythm. Echocardiography was performed using a GE Vivid 5 in accordance with international guidelines. Laboratory tests were taken in the morning in the supine position after at least 8 h of fasting.

According to laboratory results, various inflammatory markers and nutritional status assessment tools were calculated. Parameters used to assess systemic inflammation include: monocyte–lymphocyte ratio (MLR), platelet–lymphocyte ratio (PLR) and neutrophil–lymphocyte ratio (NLR), as well as the more comprehensive indices systemic inflammation response index (SIRI = neutrophils × monocytes/lymphocytes) and systemic immune–inflammation index (SII = platelets × neutrophils/lymphocytes). Nutritional indices included Confirmed Nutritional Status (CONUT), Hemoglobin–Albumin–Lymphocyte–Platelet score (HALP), Geriatric Nutrition Risk Index (GNRI), and Prognostic Nutrition Index (PNI). In addition, heart rate, PR segment duration, QT/QTc, and QRS durations were obtained from electrocardiographic analyses. ICEB was calculated as the QT/QRS duration, and its corrected form, ICEBc, is calculated as the QTc/QRS duration [14].

### 2.3. Statistical Analysis

Statistical analyses were performed using IBM SPSS Statistics version 27 (IBM Corp., Armonk, NY, USA) and R software (version 4.5.2; R Foundation for Statistical Computing, Vienna, Austria).The Kolmogorov-Smirnov test and histograms were used to assess the distribution. Data were presented according to distribution characteristics; for continuous data, normally distributed variables were shown with mean (SD), and those not normally distributed with median (IQR). Categorical variables were presented as numbers (percentages). For comparisons between groups, one-Way ANOVA was used to analyze normally distributed continuous variables, and the Kruskal–Wallistest was used to evaluate variables that did not exhibit a normal distribution. For categorical variables, the Chi-Square test and, if necessary, Fisher’s exact test were used. Pearson correlation analysis was used to evaluate the relationships between ICEB/ICEBc and clinical, nutritional, and inflammatory markers. A four-node constrained cubic spline model was used to investigate potential nonlinear relationships between Continuous BMI and ICEB/ICEBc. In all analyses, *p*-values < 0.05 were considered statistically significant.

## 3. Results

### 3.1. Patient Characteristics

Our study population consisted of patients referred to cardiology, obesity and internal medicine outpatient clinics. The study analyses were performed with 591 patients. The exclusion criteria are outlined in Figure 1. The entire population was divided into three groups according to BMI: normal weight (BMI = 18.5–25 kg/m^2^, *n* = 91), overweight (BMI = 25–30 kg/m^2^, *n* = 122), and obese (BMI > 30 kg/m^2^, *n* = 378). Significant differences were observed between BMI groups in terms of demographic, anthropometric, laboratory, and electrocardiographic parameters. The main characteristics of the total population are shown in Table 1.

The female gender ratio was predominant in obese patients, and there was a statistically significant relationship between BMI and age. Age increased with BMI (Normal: 28.2 ± 9.5, Overweight: 35.8 ± 10.6, Obese: 34.9 ± 10.6 years; *p* < 0.001), and the percentage of women was significantly higher in the obese group (78.8%) compared to normal weight patients (72.5%) and overweight patients (31.7%) (*p* < 0.001).

Laboratory findings showed significant differences between BMI groups in terms of various nutritional and inflammation markers. Hemoglobin and hematocrit were significantly lower in obese patients (*p* < 0.001 for each). Albumin levels were also similarly lower in obese patients (*p* < 0.001). Lymphocyte count increased in correlation with BMI, while monocyte count decreased (*p* < 0.001).

HbA1c and glucose levels were significantly higher in obese patients (*p* < 0.001), which we believe may be due to early metabolic disorders.

### 3.2. Electrocardiographic Parameters and ICEB/ICEBc

The primary outcomes, ICEB and ICEBc, showed statistically significant differences among BMI categories:

In the obese group, the ICEB value (4.27 ± 0.51) was higher compared to both overweight (4.04 ± 0.53) and normal weight patients (4.22 ± 0.54) (*p* < 0.001).

A similar trend was observed for ICEBc; it was highest in obese patients (4.99 ± 0.59) and lowest in overweight patients (4.59 ± 0.58) (*p* < 0.001).

**Figure 2:**
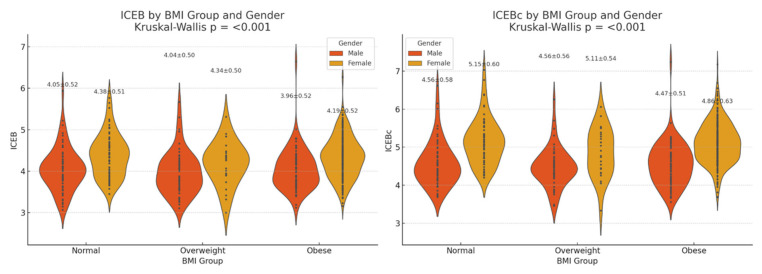
Comparison of ICEB and ICEBc values by BMI classification.

As a result of our findings, a non-linear relationship was observed, with better electrophysiological balance in the overweight group than in obese and normal weight patients. The comparison of ICEB/ICEBc values according to groups is schematically shown in Figure 2.

### 3.3. BMI Groups and Nutritional Indices

Significant differences were observed in nutritional indices among BMI groups:

GNRI was significantly higher in obese patients (138.0 ± 11.3) compared to overweight (116.7 ± 6.7) and normal weight individuals (108.3 ± 6.6) (*p* < 0.001).

HALP score was lowest in obese patients (53.6 ± 23.9) (*p* = 0.033), which we believe may be related to an underlying inflammatory event or nutritional deficiency. CONUT score was also significantly different, but overall scores were low in all groups (*p* = 0.002). In contrast, the PNI value did not show a significant difference between the groups (*p* = 0.266).

**Figure 3:**
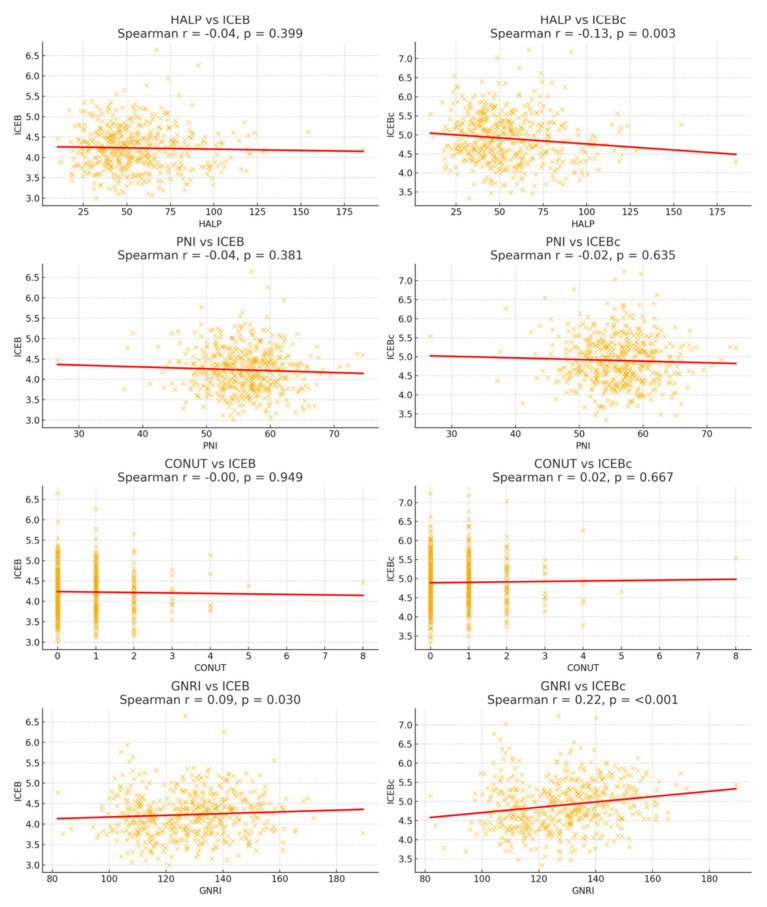
Scatter dot plots of nutritional scores across BMI groups.

**Figure 4:**
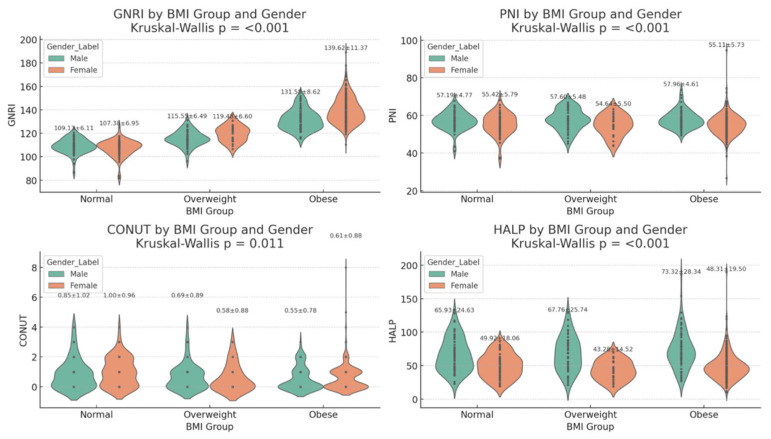
GNRI, PNI, CONUT, and HALP values stratified by BMI groups.

Scatter plots and comparative graphs (Figure 3 and Figure 4) further supported these findings by visually highlighting the degrees of nutritional scores across BMI classifications.

### 3.4. Correlation Between ICEB/ICEBc and Nutritional Parameters

Showed a positive correlation with age (r = 0.145, *p* < 0.001), a weak but significant relationship was observed. A correlation was also observed with gender (r = −0.282, *p* < 0.001), with higher ICEB values seen in women. A significant relationship was observed between ICEB and various inflammation markers. A weak but statistically significant inverse relationship was recorded with monocyte-lymphocyte ratio (MLR) (r = −0.117, *p* = 0.004) and platelet-lymphocyte ratio (PLR) (r = −0.088, *p* = 0.033). None of the nutritional indices (CONUT, HALP, GNRI, or PNI) showed a statistically significant correlation with ICEB (shown in Table 2).

ICEBc showed a significant positive correlation with BMI (r = 0.145, *p* < 0.001) and GNRI (r = 0.178, *p* < 0.001); this result suggests that ICEBc may be more sensitive to body composition and nutritional risk than ICEB itself. Analyses established with the gender variable also revealed a statistically significant relationship (r = −0.438, *p* < 0.001), supporting gender-based electrophysiological variations. Of the inflammatory markers, only MLR showed a significant negative correlation with ICEBc (r = −0.148, *p* < 0.001).

### 3.5. Nutritional Scores According to ICEB and ICEBc Quartiles

**Figure 5:**
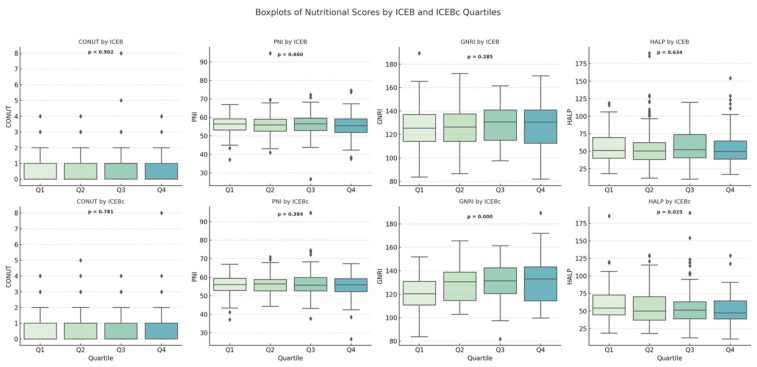
Comparison of nutritional indices across ICEB and ICEBc quartiles.

In addition to differences based on BMI, we also grouped patients according to ICEB and ICEBc quartiles to investigate possible associations with nutritional indices and compared them in terms of CONUT, PNI, GNRI, and HALP scores. Based on ICEB quarter slices, nostatistically significant differences were found between the groups in terms of CONUT, PNI, and HALP scores (*p* = 0.902, *p* = 0.660, and *p* = 0.634, respectively). Although an increasing trend was observed in GNRI levels from Q1 to Q3, this change did not reach statistical significance (*p* = 0.285). On the other hand, when the ICEBc quartiles were analyzed, significant changes were recorded, especially in the GNRI and HALP score parameters. In the analysis conducted between ICEBc quartiles, it was determined that GNRI scores showed a statistically significant upward trend from Q1 (120.9 ± 13.7) to Q4 (131.8 ± 17.6) (*p* < 0.001); conversely, HALP scores showed an inverse trend with the increase in ICEBc, reaching their lowest level in the Q4 (51.1 ± 19.4) group (*p* = 0.025). The relationship between ICEBc and GNRI suggests a possible association with nutritional status, and its relationship with HALP score suggests a possible association with inflammation. A detailed comparison of nutritional indices across the ICEB and ICEBc quartiles is presented in Table 3. It is also schematically represented in Figure 5.

**Figure 6:**
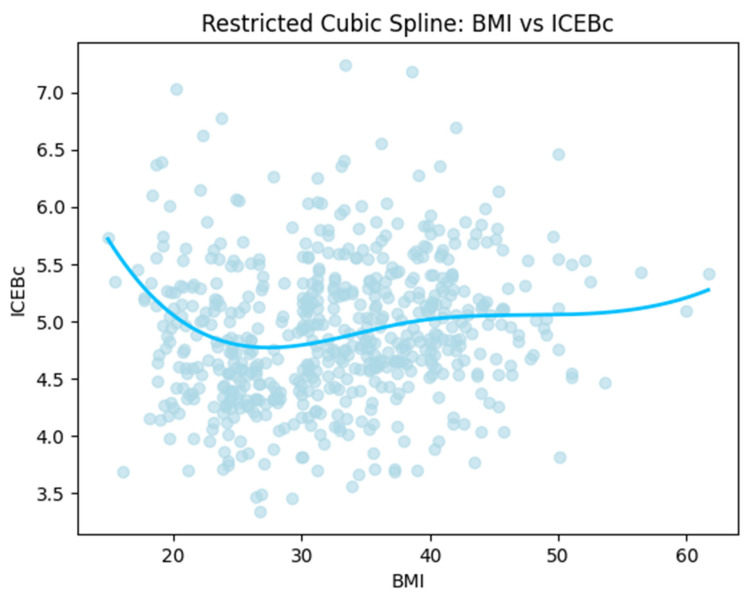
Restricted spine cubic graph.

When BMI was analyzed as a continuous variable, second-order regression models did not show a statistically significant independent second-order term for either ICEB or ICEBc. Second-order regression showed no significant association for ICEB (R^2^ = 0.001, *p* = 0.800), whereas the ICEBc model was generally significant (R^2^ = 0.025, *p* = 0.0006). Restricted cubic spline analysis revealed a significant nonlinear relationship between BMI and ICEBc in unadjusted models (R^2^ = 0.052, *p* < 0.001) (Figure 6). This relationship remained statistically significant in adjusted spline models accounting for age, heart rate, GNRI, albumin, and hemoglobin. ICEB, however, did not show a significant pattern. In multivariate regression analysis, BMI remained independently associated with ICEBc (β = −0.086, *p* < 0.001), but not with ICEB. Multivariable models were constructed, including clinically relevant covariates rather than relying solely on automated stepwise selection procedures.

## 4. Discussion

In the present study, the implications of nutritional status and inflammatory burden on the ICEB/ICEBc were evaluated in detail across different BMI categories (Normal weight, overweight, and obese groups). According to the results of our study, we found that ICEB (4.27 ± 0.51) and ICEBc (4.99 ± 0.59) values were significantly higher in obese individuals. In addition, the overweight group exhibited relatively lower ICEBc values within a U-shaped distribution. Continuous modeling using spline analysis supported a non-linear distribution pattern between BMI and ICEBc, consistent with categorical findings. In particular, ICEBc showed more significant relationships with nutritional indices such as GNRI and HALP scores compared to ICEB (shown in Table 3). Our findings suggest that ICEBc may represent a more sensitive electrophysiological marker compared to ICEB.

ICEB, BMI, and the Obesity Paradox: The majority of studies in the literature treat obesity as a monolithic risk factor that linearly increases the risk of arrhythmia [17]. Significant correlations have been identified between increased BMI and the prolongation of ventricular repolarization parameters, such as QTc and QT/QTc dispersion, indicating that obesity exacerbates repolarization abnormalities and arrhythmic risk. It has also been shown that weight loss in obese and overweight individuals leads to a reduction in QTc and QT/QTc dispersion values [18]. Supporting these findings, our study demonstrated that QTc significantly increases across normal weight, overweight, and obese individuals (410.1 ± 23.4 ms, 414.3 ± 22.4 ms, and 4.27 ± 0.51 ms, respectively; *p* < 0.001). In contrast, our findings regarding ICEB and ICEBc suggest that the relationship between BMI and cardiac electrophysiological balance does not always follow a linear trajectory. Specifically, the fact that overweight individuals demonstrated lower ICEB values compared to the normal-weight group suggests that the relationship between BMI and electrophysiological balance may not follow a strictly linear pattern. As emphasized by Lavie et al., a slight excess in body weight may play a protective role in certain cardiovascular conditions by providing metabolic reserve and autonomic balance advantages [19]. Literature data indicate that the correlation between BMI and QTc interval does not always follow a linear course; in this context, it has been reported that cardiac electrophysiological-autonomic balance exhibits a more stable character in the mildly overweight group [20]. Our findings support this non-linear relationship in the literature. In a prospective study supporting this complex interaction, the electrophysiological profiles of morbidly obese individuals undergoing bariatric surgery were examined; the analyses revealed a statistically significant, weak negative correlation between BMI and the ICEB parameter (r = −0.239, *p* = 0.004) [21].

The improvement in ventricular repolarization parameters observed alongside significant postoperative weight loss (a decrease in BMI from 45.7 to 31.7 kg/m^2^) suggests that extreme elevations in BMI may adversely impact electrophysiological balance [21]. This finding may indicate that elevated BMI is associated with alterations in electrophysiological balance.

According to the latest data in the literature, ICEB has been proposed as a potentially sensitive electrophysiological marker, with a higher sensitivity than conventional QT or QRS durations in predicting the risk of ventricular arrhythmia [5].

Previous studies have stated that an increase in ICEB leads to a prolongation of ventricular repolarization time, which in turn reflects an increased risk of Torsades de Pointes, while a decrease in ICEB reflects conduction slowing and consequently a risk of non-Torsades ventricular tachycardia [22]. In other words, it means that abnormal ICEB fluctuations provide different arrhythmic information.

The significant increase in ICEB/ICEBc observed in the obese group in our findings indicates that ventricular repolarization is relatively prolonged compared to the depolarization phase, and this may constitute an arrhythmogenic substrate. However, the potential for this electrophysiological imbalance to develop into clinical arrhythmia events can only be clarified through multicenter studies involving long-term prospective follow-up. The relatively lower ICEBc values observed in the overweight group should not be interpreted as evidence of physiological optimization or protective adaptation. Given the cross-sectional design of the study, it is not possible to determine whether these patterns represent stable physiological states, transient adjustments, or incidental findings.

Nutritional Scores and Electrophysiological Interaction: The most unique aspect of our study is the interaction of ICEBc with multidisciplinary nutritional scores such as GNRI and HALP. The relationship between nutritional status and arrhythmia has often been studied through electrolyte imbalances; however, in recent years, objective scores using more standardized nutritional indices have become prominent in examining the relationship between nutritional status and arrhythmia.

GNRI Relationship: The GNRI is a potent nutritional index that predicts mortality, particularly in elderly and chronically ill populations [23]. Numerous large cohort studies and meta-analyses have consistently shown that low GNRI values are associated with poor clinical outcomes, including cardiovascular and all-cause mortality, across various populations such as those with coronary artery disease, heart failure, and hyperlipidemia. This finding highlights that GNRI possesses significant prognostic value beyond traditional risk factors [24,25]. Our study results show that as ICEBc levels increase (as ICEBc quartiles rise), GNRI scores also increase significantly (Q1: 120.9 vs. Q4: 131.8, *p* < 0.001). However, this is because high BMI mathematically inflates the GNRI calculation; therefore, this increase is thought to reflect an increased risk of electrophysiological instability rather than a clinical ‘well-being’. This observed result demonstrates for the first time that low GNRI values may have negative effects on ventricular repolarization, extending beyond the frequently reported GNRI-mortality relationship.

HALP Relationship: The HALP score is a marker focusing on both nutrition and inflammation by combining hemoglobin, albumin, lymphocyte, and platelet values; thus, it reflects the balance between inflammation and nutrition. Uçar et al.’s study in patients with rheumatoid arthritis showed that ICEB and ICEBc were significantly higher compared to the control group (*p* < 0.001 and *p* < 0.001, respectively). Furthermore, ICEB and ICEBc were found to correlate significantly and positively with high-sensitivity C-reactive protein (hs-CRP) levels (r = 0.467, *p* < 0.001 for ICEB; r = 0.479, *p* < 0.001 for ICEBc). These findings suggest that systemic inflammation can disrupt cardiac electrophysiological balance, and as the inflammatory load increases, a pro-arrhythmic shift in the ventricular repolarization–depolarization balance may occur [26]. Cenk Soysal et al. demonstrated an inverse relationship between the HALP score and inflammation [27]. In our results, the HALP score decreased as ICEBc increased (*p* = 0.025). It is shown in Table 3. The results obtained strengthen the hypothesis that cardiac electrophysiological instability follows a course concurrently with subclinical inflammation and nutritional deficiency, and are considered to be in complete agreement with the inflammation-arrhythmia axis already defined in the literature.

CONUT and PNI: In the study by Zhang et al., high CONUT scores were significantly associated with atrial fibrillation (AF) recurrence, with higher recurrence rates observed in patients with high malnutritional risk [28]. The absence of a significant difference between CONUT/PNI and ICEB in our study suggests that these scores may be more decisive in atrial processes or acute prognosis rather than ventricular repolarization balance. As a matter of fact, patients with AF were excluded from our study.

Diversity of Parameters and Innovations: Although the impact of obesity on electrocardiographic parameters has been extensively examined, most existing reports focus primarily on traditional repolarization markers such as BMI and QT-QTc intervals; studies evaluating new electrophysiological indices in this context are relatively scarce [29]. Our study breaks this limited focus and contributes the following innovations to the literature: By using not only QT but also QRS and their ratio (ICEB/ICEBc), the balance between conduction velocity and the refractory period was analyzed. Four different nutritional scores (GNRI, HALP, PNI, and CONUT) were evaluated together in an ICEB analysis for the first time alongside BMI. Our study clearly demonstrates the superiority of ICEBc over raw ICEB values in relation to nutritional indices and inflammatory markers (such as MLR), providing a methodological guide for future research.

Although ICEB and ICEBc have shown promise as novel electrophysiological markers in recent studies, these indices remain relatively new and lack universally established reference ranges across diverse populations. Their predictive value for clinical arrhythmic events has not yet been validated in large prospective cohorts. Therefore, the present findings should be interpreted as exploratory and hypothesis-generating rather than definitive evidence of clinical risk stratification utility.

### Limitations

Our study has several important limitations. First, its retrospective and single-center nature restricts the ability to draw definitive causal conclusions and may lead to certain selection biases. The number of participants was relatively modest, and the disproportionate number of patients between study groups may have affected the overall statistical power of our comparisons. Consequently, this study should be conducted with a larger number of participants and in a multi-center format. A second important point is that our study is based only on baseline laboratory data obtained at the time of outpatient admission; long-term follow-up data are not available. This cross-sectional perspective limits our understanding of how these parameters evolve. The obesity assessment could have been more detailed. While BMI was recorded in the patient file, the lack of additional measurements, such as the waist-to-height ratio, is another shortcoming. We lack very detailed information on potential confounding factors, such as specific comorbidities and lifestyle habits, including steroid use, smoking, and alcohol consumption. All these comorbidities may have an impact on laboratory values and affect laboratory results.

## 5. Conclusions

This study demonstrates that the relationship between BMI and ICEB/ICEBc is non-linear, suggesting that a more balanced electrophysiological profile may be observed in overweight individuals. Our data reveal that obesity creates a pro-arrhythmic substrate by inducing a dominant shift in ventricular repolarization; however, this process is critically modulated by the patient’s nutritional and inflammatory profile. In particular, ICEBc stands out as a much more sensitive and clinically significant predictor compared to ICEB data, thanks to its strong correlation with multidisciplinary indices such as GNRI and HALP. This integrative approach can offer a refined and personalized methodological framework for early-stage arrhythmia risk projection in obese individuals.

## Figures and Tables

**Table 1:** Baseline characteristics of the total population.

Variable	Normal (*n* = 91)	Overweight (*n* = 122)	Obese (*n* = 378)	Total (*n* = 591)	*p*-Value
Age (years)	28.2 ± 9.5	35.8 ± 10.6	34.9 ± 10.6	33.6 ± 10.8	<0.001
Female gender, *n* (%)	66 (72.5)	26 (31.7)	298 (78.8)	391 (66.2)	<0.001
Weight (kg)	62.6 ± 10.5	78.6 ± 9.7	98.9 ± 17.3	87.6 ± 21.3	<0.001
Height (cm)	169.8 ± 8.4	170.8 ± 9.6	162.8 ± 8.8	165.7 ± 9.5	<0.001
Laboratory Findings					
Hemoglobin (g/dL)	14.5 ± 1.8	14.9 ± 1.7	13.7 ± 1.7	14 ± 1.8	<0.001
Hematocrit (%)	43.7 ± 4.9	44.9 ± 5.3	42.2 ± 4.4	42.9 ± 4.7	<0.001
WBC (10^3^/μL)	8.28 ± 2.58	8.51 ± 1.96	8.50 ± 2.38	8.45 ± 2.38	0.534
Neutrophile (10^3^/μL)	5.17 ± 2.3	5.27 ± 1.7	6.75 ± 1.9	6.20 ± 2.61	0.787
Lymphocyte (10^3^/μL)	2.29 ± 0.75	2.45 ± 0.78	2.64 ± 0.88	2.54 ± 0.85	<0.001
Monocyte (10^3^/μL)	0.52 ± 0.2	0.54 ± 0.18	0.48 ± 0.16	0.50 ± 0.17	<0.001
Platelet (10^3^/μL)	267 ± 55	281 ± 60	303 ± 73	292 ± 69	<0.001
Glucose (mg/dL)	88.1 ± 11.2	94.1 ± 16.1	94.9 ± 24	93.4 ± 21	0.008
Urea (mg/dL)	26.4 ± 7.7	28.1 ± 7.9	26.4 ± 7.2	26.6 ± 7.4	0.152
Creatinine (mg/dL)	0.75 ± 0.1	0.82 ± 0.2	0.68 ± 0.1	0.71 ± 0.2	<0.001
Uric acid (mg/dL)	4.9 ± 1.2	5.3 ± 1.2	5.4 ± 1.3	5.26 ± 1.31	0.002
Albumin (mg/dL)	44.9 ± 3.9	44.4 ± 4.1	42.5 ± 3.3	43.3 ± 3.7	<0.001
Total cholesterol (mg/dL)	188.7 ± 46.3	193.2 ± 39.5	190.0 ± 37.6	190.1 ± 40.3	0.731
LDL (mg/dL)	106.4 ± 34.4	115.8 ± 33.8	113.5 ± 30.5	112.1 ± 32	0.030
HbA1c (%)	5.30 ± 0.34	5.36 ± 0.35	5.63 ± 0.62	5.52 ± 0.56	<0.001
Electrocardiography Findings					
Heart rate (bpm)	80.5 ± 20	78.5 ± 19	83 ± 17	80.4 ± 12.2	0.008
P wave (msc)	101.2 ± 12.4	103.8 ± 12.2	103.9 ± 14.1	103.3 ± 13.5	0.123
PR Segment (msc)	137 ± 18	141.7 ± 17.6	144.6 ± 18.1	142.5 ± 18.2	<0.001
QT duration (msc)	356.9 ± 27.9	364.5 ± 29.3	359.8 ± 27.4	359.7 ± 27.8	0.118
QTc duration (msc)	410.1 ± 23.4	414.3 ± 22.4	419.8 ± 22.9	416.8 ± 23.4	<0.001
QRS duration	86.1 ± 9.8	91.4 ± 10.8	85.1 ± 9.4	86 ± 10	<0.001
ICEB	4.22 ± 0.54	4.04 ± 0.53	4.27 ± 0.51	4.23 ± 0.52	<0.001
ICEBc	4.87 ± 0.66	4.59 ± 0.58	4.99 ± 0.59	4.91 ± 0.62	<0.001
Nutritional Assessment Tools					
CONUT	1 (2)	0 (1)	0 (1)	0 (1)	0.002
HALP	57.9 ± 23.3	60.3 ± 25.5	53.6 ± 23.9	49.3 (24)	0.033
GNRI	108.3 ± 6.6	116.7 ± 6.7	138.0 ± 11.3	128.1 ± 16.4	<0.001
PNI	56.3 ± 5.4	56.7 ± 5.6	55.7 ± 5.6	56 ± 5.6	0.266

**Table 2:** Correlation between ICEB/ICEBc and other parameters.

ECG Parameters	Variable	Correlation (r)	*p*-Value
ICEB	PNI	−0.046	0.266
	GNRI	0.054	0.210
	HALP	0.074	0.075
	CONUT	−0.011	0.800
	BMI	0.023	0.578
	Waist/height ratio	0.067	0.305
	Age	0.145	<0.001
	Gender	−0.282	<0.001
	NLR	−0.06	0.147
	PLR	−0.088	0.033
	MLR	−0.117	0.004
	SII	−0.059	0.151
	SIRI	−0.063	0.128
ICEBc	PNI	−0.037	0.379
	GNRI	0.178	<0.001
	HALP	0.064	0.025
	CONUT	0.019	0.650
	BMI	0.145	<0.001
	Waist/height ratio	0.115	0.079
	Age	−0.004	0.927
	Gender	−0.438	<0.001
	NLR	−0.043	0.303
	PLR	−0.019	0.639
	MLR	−0.148	<0.001
	SII	−0.038	0.357
	SIRI	−0.049	0.24

**Table 3:** Comparison of nutritional indices across ICEB and ICEBc quartiles.

**ICEB**					
**Nutritional Indices**	**Q1 (3.00–3.85)**	**Q2 (3.85–4.21)**	**Q3 (4.21–4.54)**	**Q4 (4.54–6.65)**	***p*-Value**
CONUT	0.66 ± 0.9	0.67 ± 0.9	0.73 ± 1	0.66 ± 0.8	0.902
PNI	56.1 ± 5.1	56.1 ± 5.9	56.3 ± 5.8	55.5 ± 5.5	0.66
GNRI	126.3 ± 16.8	127.1 ± 16.1	129.7 ± 15.6	128.8 ± 16.5	0.285
HALP	56 ± 23	55.3 ± 27.3	57.2 ± 23	53.6 ± 22.4	0.634
**ICEBc**					
**Nutritional Indices**	**Q1 (3.34–4.50)**	**Q2 (4.50–4.86)**	**Q3 (4.86–5.29)**	**Q4 (5.29–7.24)**	***p*** **-Value**
CONUT	0.68 ± 0.9	0.7 ± 0.9	0.62 ± 0.8	0.72 ± 1.0	0.781
PNI	55.9 ± 5.1	56 ± 5	56.6 ± 6.5	55.4 ± 5.6	0.384
GNRI	120.9 ± 13.7	129 ± 15.1	130.5 ± 16.3	131.8 ± 17.6	<0.001
HALP	59.7 ± 24.4	56.1 ± 25.3	55.2 ± 25.9	51.1 ± 19.4	0.025

ICEB: Index of Cardiac Electrophysiological Balance, CONUT: Controlling Nutritional Status, PNI: Prognostic Nutritional Index GNRI: Geriatric Nutritional Risk Index, HALP: Hemoglobin–Albumin–Lymphocyte–Platelet Score, ICEBc: Corrected Index of Cardiac Electrophysiological Balance. Q1–Q4 represent quartiles of ICEB (Q1: 3.00–3.85; Q2: 3.85–4.21; Q3: 4.21–4.54; Q4: 4.54–6.65) and ICEBc (Q1: 3.34–4.50; Q2: 4.50–4.86; Q3: 4.86–5.29; Q4: 5.29–7.24) values.

## Data Availability

The original contributions presented in this study are included in the article. Further inquiries can be directed to the corresponding author.

## Abbreviations

CVD	Cardiovascular Diseases
ICEB	Index of Cardiac Electrophysiological Balance
ICEBc	Corrected Index of Cardiac Electrophysiological Balance
QT interval	Reflecting Ventricular Repolarization
QTc interval	Corrected Reflecting Ventricular Repolarization
QRS duration	Representing Ventricular Depolarization
BMI	Body Mass Index
GNRI	Geriatric Nutritional Risk Index
HALP	Hemoglobin–Albumin–Lymphocyte–Platelet
CONUT	Controlling Nutritional Status
PNI	Prognostic Nutritional Index
ECG	Electrocardiography
NLR	Neutrophil–Lymphocyte Ratio,
PLR	Platelet–Lymphocyte Ratio
MLR	Monocyte–Lymphocyte Ratio
SII	Systemic Immune–Inflammation Index
SIRI	Systemic Inflammation Response Index
HbA1c	Glycated Hemoglobin
VT	Ventricular Tachycardia
CRP	C-Reactive Protein
AF	Atrial Fibrillation
